# Psychological maltreatment and its relationship with self-esteem and psychological stress among adolescents in Tanzania: a community based, cross-sectional study

**DOI:** 10.1186/s12888-019-2139-y

**Published:** 2019-06-11

**Authors:** Adela A. Mwakanyamale, Yu Yizhen

**Affiliations:** Department of surgical and medical nursing, Hubert Kairuki Memorial University, Dar es salaam, P.o Box 65300 Tanzania

**Keywords:** Psychological maltreatment, Self-esteem, Psychological distress, Adolescents, Tanzania

## Abstract

**Background:**

Despite the growing recognition of childhood psychological maltreatment as a public health and human rights concern, it remains rampant in developing countries including Tanzania and has a negative impact on the victim’s self-esteem during adolescence. There is a lack of published studies in Tanzania that examine the relationship between childhood psychological maltreatment and self-esteem during adolescence. This study describes the relationship between childhood psychological maltreatment and self-esteem and psychological distress among adolescents in Tanzania.

**Methods:**

This was a cross-sectional, community-based study of secondary school students that was conducted in randomly selected secondary schools in five regions in Tanzania between April 2016 and February 2017. A multistage cluster sampling technique was employed to obtain the required number of study participants. The Rosenberg self-esteem scale, Kessler psychological distress scale (K10) and Childhood Trauma Questionnaire (CTQ) questionnaires were used to measure the variables in the study. Pearson correlation analysis was used to analyse the correlation between variables (Psychological maltreatment and self-esteem and psychological distress).

**Results:**

A sample of 1000 secondary school students was recruited for this study, of which 553 (55.3%) were males and 447 (44.7%) were females. The mean age at presentation was 16.45 ± 6.42 years**.** Out of the 1000 participants, 766 (76.6%) experienced psychological maltreatment. Emotional abuse was reported in 24.7% of the participants, while emotional neglect was reported in 51.9% of cases. There was a strong positive correlation between psychological maltreatment and self-esteem (*r* = 0.55, *p* < 0.001), whereas the correlation between psychological maltreatment and psychological distress was significantly but weak (*r* = − 0.086, *p* = 0.007). The results also show a strong positive correlation between psychological distress and self-esteem (*r* = 0.16, *p* < 0.001).

**Conclusion:**

Finding from this study demonstrated that childhood psychological maltreatment is prevalent in our setting and is associated with psychological distress and low self-esteem during adolescence. Urgent intervention targeting at reducing occurrence of childhood psychological maltreatment is necessary to reduce the incidence of low self-esteem and psychological distress among Tanzanian adolescents.

## Background

Psychological maltreatment is the most prevalent form of child abuse and is increasingly recognized as an essential component of child maltreatment and the unifying concept that connects cognitive, affective, and interpersonal problems related to physical abuse, sexual abuse, and neglect [1]. Psychological maltreatment involves repeated interactions between a parent and child that are often verbal in nature that negatively affect the emotional, social, cognitive or even physical development of a child [2]. These interactions typically include acts of commission as well as omission, such as spurning, terrorizing, isolating, exploiting and denying emotional responsiveness [3].

Psychological maltreatment is believed to negatively affect the emotional, social and cognitive development of a child, making them more prone to substance abuse and other forms of psychopathology [4]. Psychological maltreatment is thought to be the most common form of maltreatment and encompasses both emotional abuses as well as emotional neglect and also tends to accompany other forms of maltreatment, such as physical and sexual abuse [5].

Emotional abuse refers to verbal assaults on a child’s sense of worth or well-being, or any humiliating, demeaning, or threatening behaviour directed toward a child by an older person [6].

Emotional neglect is defined as failure of caretakers to provide a child’s basic psychological and emotional needs, such as love, encouragement, belonging and support [7]. Emotional abuse can hurt to the same amount as physical abuse as it is not easy to identify, given that its effect may not be seen physically since the marks are on the inside instead of the outside [7] .It is commonly understood that there exist a few well designed and approved measures of childhood emotional abuse. Children with emotional child abuse become extremely loyal to their parents because of the fear of being punished in cases where they report abuse or think that this type of abuse or have a feeling that this is part of the normal way of life [8].

Indications of emotionally abused child include unsuitable way of behaving that may be associated with actions showing immaturity or more mature in relations to the child’s age [9]. Overall change in behavior distraction of activities, clinging or impulsively seeking affection and attention forcefulness, unsupportiveness, bedwetting or loss of ability for bowel control (after a child has been trained), and damaging or disruptive behavior, constantly being sad and withdrawn) [10]. Moreover, this may also be indicated with poor relationships with age group peers, lacking of self-confidence and self-assurance, uncommon fears for the child’s age such as fear of going home, being left alone, specific objects, or inability to react with emotion or develop an emotional bond with others, are also indicators. It is important to understand that any of the above behaviors may also be seen in normal children, but a change in pattern of these behaviors is a strong indicator of emotional abuse [11]. Emotional abuse and physical abuse may occur and may be induced by the same reasons, parents may become susceptible to being involved in the maltreatment in cases where stress in their lives have circumstances associated with stress in their lives or stress levels that are way up beyond their control [12].

Such dispositions in parents may lead to decreased capacity for consideration and handling of their children affairs as expected of them which may result in slowed mental growth or mental retardation, psychopathology, alcoholism, drug abuse and unrealistic ideas about their children’s needs or sadistic psychosis [13].

It also occurs that the abuser’s goal may be to control. Nevertheless, a single factor may not lead to abuse, but a collection of many factors may lead to creation of social and emotional burdens that may result in emotional abuse [14].

The known types of problems that can contribute to emotional abuse by parents may be related to social problems that can result in family stress such as joblessness, scarcity or poverty, segregation from relatives and friends, separation, death, adolescent parents, health crises, illness of a family member, incapacity of one of the family members or even drug and alcohol abuse within the family and mental health problems (mental incapacity, depression) [15].

The implications of emotional child abuse can be grave and long-standing. A number of research studies have concluded that psychopathologic signs are more likely to result in children who are emotionally abused. As a result, children with this effect may experience long-term depression, disaffection, anxiety, low self-esteem and unstable relationships or failure to show empathy to their counterparts [16]. During their childhood, victims of abuse may fail to flourish or their growing progress may be halted or slowed. Some may find it difficult to adjust emotionally and psychologically [17].

Some adolescents often find it difficult to trust, participate and a get the happiness in their relationships with others as a result of the abuse during childhood. Later in adulthood they may have trouble knowing and appreciating the needs and feelings of their own children and end up emotionally abusing them as well [18].

**Emotional Neglect** comes as a result of a parent’s inability to respond well *enough* to a child’s emotional needs, sometimes *associated to* mistreatment and abuse. Whereas mistreatment and abuse are parental *acts*, emotional neglect is a parent’s *failure to act.* It’s a failure and sometimes inability to notice, attend to, or responds appropriately to a child’s feelings. Given that it is an *act of omission*, it’s not easily visible, noticeable or memorable [19].

**Neglect** is the white space in the family picture, the background rather than the foreground. As much as it is often overlooked it does silent harm and damage to the concerned children [20]. Emotionally neglected children in turn grow up to a set of struggles. Since their emotions were not made valid by their parents in adulthood, they are likely to face a struggle identifying their own emotions. Understanding their own feelings as well as those of others may even be more difficult, this is because an important part of themselves and their emotional self has been denied and this may lead to a feeling of disconnect, unfulfillment or emptiness [21]. Trusting and relying upon others becomes even difficult to them, sometimes they may have a feeling of being different from others and a feeling that something that is wrong but do not know what it is that is wrong [22].

In another way parents can emotionally neglect their children without knowing what damage they are causing to their children. Being neglected may lead to struggle with self-disciplines in adulthood.

It occurs that at the end, most often during adulthood those who experienced neglect do not have the childhood memories and therefore their difficulties are blamed on themselves rather than on their parents’ failures [23].

Today emotional neglect has been ignored. This is because it’s not easily noticed and cannot be recalled by its victims. It has in many ways been overshadowed by the more visible childhood events, such as abuse and trauma [23].

Emotional abuse and neglect have been reported to cause significant harm to the child’s development, and this harm extends into adolescence and adult life [24]. Unlike cases involving physical or sexual abuse, emotional abuse and neglect are believed to be the least likely to be reported by victims; victims are rarely able to demonstrate visible proof and therefore lack a sense of legitimacy [25]. Meta-analyses on the prevalence of different types of maltreatment have reported 17.7% for physical abuse, 26.7% for psychological abuse, 11.8% for sexual abuse and 16.3% for neglect [26].

Globally, the prevalence of psychological maltreatment has been estimated to be 36% of children [26]. One study in Turkey found that approximately 51% of children were psychologically maltreated [27]. According to agency reports, the prevalence of psychological maltreatment in England, the USA and Canada ranged from 11 to 34% [28]. A high prevalence ranging from 31.3 to 68.5% has been reported in East Asia and the Pacific Region [29].

Previous studies e.g., [30, 31] have reported a strong positive correlation between psychological maltreatment and low self-esteem.

This correlation between low self-esteem and psychological maltreatment is derived by the fact that psychological maltreatment in children can negatively affect the cognitive, social and emotional development of a child.

Self-esteem can be explained as continued self-evaluation and self-belief that one is strong, worthy, famous and successful [32]. Individuals with high self-esteem feel quite positive about their characteristics and competencies, which can positively influence well-being, while low self-esteem can lead to many emotional and behavioural problems [33].

Many studies have also evaluated the relationship between self-esteem and emotional and behavioural problems [34] . Findings from these studies suggest that individuals with more emotional and behavioural problems have lower self-esteem. Parents play a keyhole in the development of self-esteem, which reflects individuals’ evaluations of themselves and their competencies [35]. Psychological maltreatment experiences include parental acts that negatively affect mental health and development [36]. Psychological maltreatment is also associated with psychological distress, which is largely defined as a state of emotional suffering characterized by symptoms of depression (e.g., lost interest; sadness; hopelessness) and anxiety (e.g., restlessness; feeling tense) [37]. Psychological distress is reported to be an indicator of the mental health of the population in public health [38].

The psychological maltreatment of children is widespread in developing countries including Tanzania and is believed to affect the overall psychological adjustment of the child [10]. As a result, victims may experience psychological distress, with symptoms of low self-esteem, depression and anxiety that may result in substance abuse [39].

However, it has been suggested that in a cultural environment where harsh disciplining techniques are accepted, the adverse impact on recipients may be minimal [40].

Studies conducted in sub-Saharan countries have reported that many children are exposed to high levels of psychological maltreatment [41]. Experiences from Tanzania show that parents often employ harsh physical and emotional discipline practices and believe that they do not harm their children [42].

Several studies conducted in most developing countries show a strong relationship between exposure to psychological maltreatment and low self-esteem [43]. Psychological maltreatment, including emotional abuse and neglect, is extremely common in Tanzania, yet not as broadly researched as sexual and physical abuse [44].

Decades of research on psychological maltreatment in childhood have resulted in publications on the subject in the developed countries, especially the U.S. and Europe. To our knowledge, no research or publication has been done on the relationship between psychological maltreatment and self-esteem in childhood in many developing countries. Studies have shown that witnessing domestic violence has a negative impact on a child’s well-being and healthy development, especially in relation to psychological aspects such as low self-esteem [45].

This study aims to cover that knowledge gap and address the relationship between self-esteem and psychological maltreatment in childhood in developing countries. We conducted our study in five different cities in Tanzania, East Africa.

## Methods

### Study design

A cross-sectional, community-based study was conducted at randomly selected secondary schools in Tanzania between April 2016 and February 2017.

### Study participants

Approximately 1115 secondary school students received the questionnaires and were asked to return the completed questionnaires in sealed envelopes. A total of 1000 secondary students returned the completed materials (89.7% response rate), of which 553 (55.3%) were male and 447 (44.7%) were female, as shown in Table 1. The participants’ ages ranged between 13 and 21 years old.

**Table 1:** Age group and gender distribution

	Age of the participants			Total
	13–15	16–18	19–21	
Gender				
Male	211	282	60	553
Female	83	259	105	447
Total	294	541	165	1000

### Measures

The Rosenberg self-esteem scale, Kessler psychological distress scale (K10) and Childhood Trauma Questionnaire were used to measure the variables in this study.

Childhood self-esteem was assessed using the Rosenberg Scale, a 10-item scale. Self-esteem can be defined as positive and negative feelings about oneself. In the Rosenberg Self-Esteem Scale [46], high scores are indicative of positive self-regard, while the lower scores indicate negative self-regard. We also used a 4-point Likert scale to rate the Rosenberg Self-Esteem items (1 = strongly agree, and 4 = strongly disagree). The final scores were determined by summing all the ratings from the Likert scale. A final score between 15 and 20 was indicative of normal self-esteem, a final score < 15 indicate low self-esteem, while a final score < 25 indicate high self-esteem. The Rosenberg Self-Esteem Scale is used to estimate self-esteem globally, with a high level of reliability and validity [46], and the scale has a test-retest correlation between 0.82 and 0.88.

#### The childhood trauma questionnaire (CTQ)

The Childhood Trauma Questionnaire (CTQ) was established from a 70-item retrospective questionnaire in which participants were required to rate the frequency (0- never true to 5- very often true) of abuse and neglect events that they had experienced during their childhood.

The CTQ was established by Bernstein and Fink. The CTQ is a self-reported inventory providing reliable and effective screening for a history of childhood neglect and abuse [47].

The CTQ is also appropriate for adolescents (age 12 and over) [48]. CTQ items assess exposure to 10 types of ACEs, including abuse (i.e., physical, emotional and sexual), neglect (i.e., emotional and physical), and household challenges (i.e., mental illness, substance abuse, physical violence, parental divorce and incarcerated family members) prior to age 18. Children are required to answer a series of statements regarding childhood life experience that are endorsed on a 5-point Likert scale according to their frequency [49].

The CTQ is psychometrically comprehensive in community samples, with the best test-retest reliability [50], displaying convergent and discriminant validity [50]. It has been demonstrated that the CTQ can display test-retest reliabilities between 0.79 and 0.86 and internal consistency reliability ranging from 0.66 to 0.92 [51].

For the purpose of this study, we used seven items in the CTQ questionnaire. The seven items focused on psychological maltreatment. Three of them measured emotional neglect, while the other four also assessed emotional abuse.

The Kessler Psychological Distress scale (K10) was developed in 1992 by Professors Kessler and Mroczek. It is globally used to measure non-specific psychological distress in the anxiety-depression spectrum. It was used in this study to assess psychological disorders, as most of these disorders are associated with anxiety and depressive symptoms [52]. The Kessler Psychological Distress scale has been used worldwide in WHO international studies and is also identified as a cross-cultural scale. A high score in the Kessler Scale is indicative of a psychological disorder [52].

The K10 comprises ten questions about psychological distress, and items are rated on a five-point ordinal scale. The total K10 score for each respondent was calculated by summing all 10 items, and scores ranged from 10 to 50.

A score under 20 is considered normal (no stress), a score of 20–24 indicates a mild mental disorder (mild stress), a score of 25–29 indicates a moderate mental disorder (moderate stress), and a score of 30 and over indicates a severe mental disorder (severe stress) [52].

### Sampling technique

A multistage cluster sampling technique was employed to obtain the required number of study participants. A sampling frame from the list of secondary school students from randomly selected secondary schools in five regions in Tanzania was prepared and used to draw up the sample. From the sampling frame, study units were sampled through the simple random method until the required sample was obtained.

### Study variables

There were two major variables in the study, including independent and dependent variables. The independent variable represents psychological maltreatment, while self-esteem and psychological stress were treated as the dependent variables in this study.

### Statistical data analysis

The statistical data analysis was performed using the Statistical Package for Social Sciences (SPSS) version 21.0 for Windows (SPSS, Chicago IL, USA). The descriptive and inferential statistics were calculated (mean (M), SD = Standard deviation, P = *p*-value) and ranges were calculated for continuous variables, whereas proportions and frequency tables were used to summarize categorical variables. Pearson correlation analysis was conducted to examine the relationship between variables, which describes the direction and strength of a relationship between two continuous or interval variables in our study, where the direction can be negative or positive.

In our study, psychological maltreatment was treated as a predictor variable, while both self-esteem and psychological stress was treated as dependent variables. A *p*-value of 0.001 was considered to be significant.

#### Ethical consideration

Ethical clearance was obtained from the review committee from the Huazhong University of Science and Technology, Tongji Medical College, China and the Ministry of Education in Tanzania. A formal letter was submitted to the respective educational district offices and subsequently to head teachers of secondary schools where the study was conducted. Written permission from the parents of the respondents was obtained a day before the time of data collection. Oral and written permission from the schools and the respective study subjects was obtained. The study was explained to the subjects. Its purpose and its confidentiality were explained, and the subjects’ consent to participate in the study was assured before completing the questionnaire.

## Results

### Social-demographic characteristics

Out of the 1000 participants in the present study, 553 (55.3%) were males and 447 (44.7%) were females. The male-to-female ratio was 1.2:1. The ages of the participants at presentation ranged from 13 to 21 years with a mean of 16.45 ± 6.42 years. The majority of participants were in the age group of 16–18 years, accounting for 541 (54.1%) cases (Table 1).

Table 2 shows that out of the 1000 participants, 766 (76.6%) experienced psychological maltreatment. Emotional abuse was reported in 24.7% of the participants, while emotional neglect was reported in 51.9% of cases. Comparing males and females, the prevalence of emotional abuse was higher in female participants (26.2%) than their male (23.5%) counterparts, while the prevalence of emotional neglect was higher among male participants (53.3%) than female (50.1%) participants. Regarding age group distribution, the prevalence of emotional abuse (30.5%) and emotional neglect (55.8%) was higher in the 19–21-year age group than the other age groups.

**Table 2:** Prevalence of psychological maltreatment by gender and age group

	Total	Emotional abuse	Emotional neglect
	*N* = 1000	247 (24.7%)	519 (51.9%)
Age			
13–15	294 (29.4)	66 (22.4)	144 (49.0)
16–18	541 (54.1)	131 (24.2)	283 (52.3)
19–21	165 (16.4)	50 (30.5)	92 (55.8)
Gender			
Male	553 (55.3)	130 (23.5)	295 (53.3)
Female	447 (44.7)	117 (26.2)	224 (50.1)

Table 3 shows severe psychological distress, reported in higher 71(54.6%) rate among female students than male 59(45.4%). However, the difference was not statistically significant *P-value = 0.072.*

**Table 3:** Prevalence of psychological distress by gender

Psychological Distress	Sex N (%)		*P-Value*
	Male	Female	
Normal	332 (56.0)	261 (44.0)	0.072
Mild	105 (59.3)	72 (40.7)	
Moderate	59 (59.0)	41 (41.0)	
Severe	59 (45.4)	71 (54.6)	

The findings in Table 4 show a strong positive correlation between psychological maltreatment and self-esteem (*r* = 0.51, *p* < 0.001), whereas the correlation between psychological maltreatment and psychological distress was significantly but weak (*r* = − 0.086, *p* = 0.007). The results also show a strong positive correlation between psychological distress and self-esteem (*r* = 0.16, *p* < 0.001).

**Table 4:** Correlation between psychological maltreatment, self-esteem and psychological distress

Variables	M ± SD	Self-esteem	Psychological distress	Psychological maltreatment
Self-esteem	27.6 ± 4.2	1.00		
Psychological distress	24.6 ± 4.5	.164^**^	1.00	
Psychological maltreatment	10.4 ± 2.3	.515^**^	−.086^**^	1.00

^**^. Correlation is significant at the 0.01 level (2-tailed)

^**^p < .001. M: Mean, SD = Std Deviation, *N* = 1000 (Number of participants)

Finally, the linear regression model (Table 5) was calculated to determine the impact of psychological maltreatment to both low self-esteem and psychological distress.

**Table 5:** Linear regression analysis on self-esteem and psychological distress

Variables	Unstandardized B	Standardised Coefficient Beta(β)	Standard Error
*Predicted variable Self –esteem*			
Intercept	18.09^**^		.516
Psychological Maltreatment		.515^**^	.50
R Square	.265		
F (1,998)	359.89		
*Predicted variable: Psychological distress*			
Intercept	26.34^**^		
Psychological Maltreatment		-. 086^**^	.642
R Square	.007		.06
F (1,998)	7.36		

*N* = 1000

^**^p < 0.01

The regression result reveals that psychological maltreatment has a statistically significant impact on self-esteem, *R*^*2*^ = .26, β = .515, F (1,998) =35.8, *p* < .01.

Approximately 26% of the variance in self-esteem can be explained by psychological maltreatment. Moreover, psychological maltreatment has also a statistically significant impact on psychological distress *R*^*2*^ = .007, β = −.086, F (1,998) =7.36, *p* < .01. This means that the value of 0.6% of the variance in psychological distress can be explained by one-unit change in psychological maltreatment.

## Discussion

Psychological maltreatment in children represents the most challenging and prevalent form of child abuse and neglect worldwide [53], but until recently, it has received relatively little attention in developing countries such as Tanzania. In this study, the presence of psychological maltreatment during childhood was reported in 76.6% of cases, emotional abuse was reported in 24.7% of the participants, while emotional neglect was reported in 51.9%, which is comparable with that reported in developing countries [54] but higher than that observed in many other studies in developed countries [55]. The prevalence of psychological maltreatment in the present study may be underestimated because this form of child maltreatment is mostly underreported to authorities for fear of being arrested by the police. Also, psychological maltreatment is often not recognized when other forms of maltreatment, such as physical and sexual abuse, coexist [56]. The prevalence rates of psychological maltreatment may also be underestimated because they capture a wide range of parenting behaviours, and there is little to no consensus across studies as to what phenomena should be included [57].

Psychological maltreatment consists of both emotional abuse as well as emotional neglect [58, 59]. In the present study, the prevalence of emotional neglect was higher among male participants than female participants, this finding agrees with what has previously been reported [60], emotional abuse was reported in 24.7% of the participants, while emotional neglect was reported in 51.9% of cases. The prevalence of emotional abuse was higher among female participants than their male counterparts. The reason for the underlying vulnerabilities and gender influence on children with emotional abuse and neglect is unclear, and this requires further investigation.

Although psychological maltreatment is the most common form of childhood maltreatment in developing countries such as Tanzania, no participants who were psychologically maltreated sought professional treatment. This observation may be attributed to the lack of awareness and poor knowledge regarding which childhood experiences constitute psychological maltreatment. This calls for urgent preventive measures to reduce the occurrence of childhood psychological maltreatment so as to reduce the potential adverse consequences of psychological maltreatment that occurs during adolescence and adulthood.

Psychological maltreatment is strongly associated with low self-esteem in adolescents [16]. This finding agrees with our study, which found that childhood psychological maltreatment was statistically significantly associated with self-esteem during adolescence. Parenting style plays a crucial role in a child’s social, cognitive and emotional development. A negative parenting style involving psychological maltreatment may result in low self-esteem in adolescents that continues to adulthood [17].

The results reported here agree with previously published studies, demonstrating correlation between childhood exposure to psychological maltreatment and self-esteem. Children exposed to high levels of psychological maltreatment have been reported to show low level of self-esteem during adolescence. For a psychologically maltreated child, the negative impacts are believed to occur when the child internalizes the harsh treatment, including the negative messages from parents and/or care givers [18]. This results in insecurities, and the child develops maladaptive interpersonal schemas, which lead to dysfunctional behaviours [61].

Children in such situations tend to believe that they are worthless and that the world is unsafe and perceive everybody as abusive [19]. Those with low self-esteem tend to be more self-conscious and isolate themselves from others [62].

There are some limitations of the study that should be noted: first, the cross-sectional study design does not allow for the establishment of a causal relationship; therefore, only the association between psychological maltreatment and self-esteem was established in this study.

Second, the study participants included secondary school students from randomly selected secondary schools in five regions in Tanzania; therefore, the results cannot be generalized to the Tanzanian population as a whole. Third, additional consideration should be given to the tendency of participants to under- or over-report incidents of maltreatment. Thus, the results must be interpreted with caution.

## Conclusion

The purpose of this study was to build upon the extant literature concerning childhood psychological maltreatment and its relationship to self-esteem and psychological distress. The results demonstrate that childhood psychological maltreatment is prevalent in our setting and is statistically significant because it causes the reduction of self-esteem among Tanzanian adolescents. Moreover, exposure to psychological maltreatment during childhood was found to be significantly weak associated with an increased likelihood of experiencing psychological distress in adolescence. The understanding of the interrelatedness of psychological maltreatment with self-esteem should be considered in the design of studies, treatments, and programs to prevent psychological maltreatment as well as low self-esteem.

Urgent preventive measures aiming at reducing the incidence of childhood psychological maltreatment is necessary to lessen the incidence of low self-esteem and psychological distress among Tanzanian adolescents. Strategic national educational programs should be implemented to bring awareness of the adverse effects of childhood psychological maltreatment. Parents, caregivers and teachers will benefit from realizing the effects of childhood psychological maltreatment and be encouraged to take an alternative method of discipline.
